# Trimethylamine N-Oxide Promotes Abdominal Aortic Aneurysm Formation by Aggravating Aortic Smooth Muscle Cell Senescence in Mice

**DOI:** 10.1007/s12265-022-10211-6

**Published:** 2022-02-10

**Authors:** Jiaxin Hu, Jiamin Xu, Song Shen, Wengfeng Zhang, Haiting Chen, Xuan Sun, Yu Qi, Ying Zhang, Qi Zhang, Meng Guo, Ningxin Peng, Biao Xu

**Affiliations:** 1 Department of Cardiology, Nanjing Drum Tower Hospital, The Affiliated Hospital of Nanjing University Medical School, MOE Key Laboratory of Model Animal for Disease Study, Nanjing University, Zhongshan Road, Nanjing, 210061 China; 2 Department of Cardiology, Nanjing Drum Tower Hospital Clinical College of Nanjing Medical University, Nanjing, China

**Keywords:** TMAO, Abdominal aortic aneurysm, VSMC, Senescence, AngII, CaCl_2_

## Abstract

Trimethylamine N-oxide (TMAO) has been linked to cardiovascular disease morbidity and mortality. However, the role of TMAO in the development of abdominal aortic aneurysms (AAAs) is not known. This study investigated the association between TMAO and AAA formation. TMAO and saline were added to the drinking water of angiotensin II (AngII)- and calcium chloride (CaCl_2_)-induced AAA model mice, respectively. After 4 weeks, the effects of TMAO on AAA development were determined by histology and immunohistology of aortic tissue. The in vitro effects of TMAO were also examined in mouse aortic smooth muscle cells (SMCs). The maximal aortic diameter, incidence of AAA, and degree of elastin degradation were significantly increased in TMAO-treated mice. TMAO also increased the accumulation of the senescence markers p21 and p16, as well as of reactive oxygen species (ROS), matrix metalloproteinase-2 (MMP2), and matrix metalloproteinase-9 (MMP9) in vivo and in vitro. TMAO promoted AAA development in mouse AAA models induced by AngII and CaCl_2_ by a mechanism involving cellular senescence.

**Graphical abstract:**

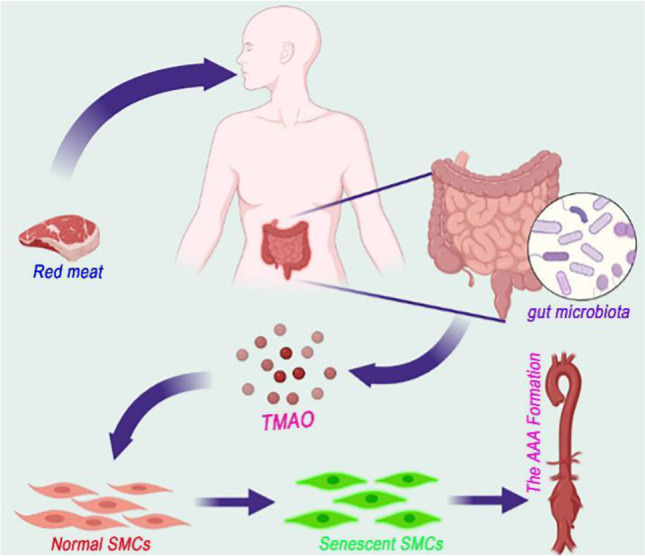

## Introduction

The term abdominal aortic aneurysm (AAA) describes a localized dilatation of the artery that exceeds 50% of its normal diameter [1]. The prevalence and mortality rate increase with age. Epidemiology shows that 1.3% of all deaths in men between the ages of 65 and 85 in developed countries are caused by AAA ^2^. The pathology of AAA is characterized by degradation of the extracellular matrix, oxidative stress, vascular inflammation, and senescence of vascular smooth muscle cells [1–4]. Because the pathogenesis of AAA is elusive, the treatment of AAA currently relies on surgical repair, and no effective drug therapy is available.

Trimethylamine N-oxide (TMAO), a metabolite produced by gut microorganisms, is a dose-dependent and independent risk factor for cardiovascular disease (CVD) [5]. Atherosclerosis is a major component of cardiovascular disease. TMAO has been shown to contribute to atherosclerosis and is highly associated with CVD risk [6–8]. AAA shares the same risk factors as atherosclerosis, and vascular inflammation plays a vital part in the development of AAA. TMAO induces smooth muscle cell calcification and vascular senescence through increased vascular inflammation [9, 10]. This evidence suggests that TMAO may promote the development of AAA, but at present, no clear link has been found between TMAO and AAA development. This study demonstrated for the first time that TMAO can promote the development of AAA and that the mechanism may be related to TMAO-induced senescence of smooth muscle cells.

## Methods

### Mouse Experimental Protocol

All animal protocols were approved by the Institutional Ethics Committee of Nanjing Drum Tower Hospital (2019AE01062) and conducted following the guidelines outlined in the Guide for the Care and Use of Laboratory Animals published by the National Institutes of Health (Eighth Edition). Eight-week-old C57BL/6 mice were obtained from the Model Animal Research Center of Nanjing University, while apoe-/- C57BL/6 mice were obtained from the Jackson Laboratory (Bar Harbor, ME, USA). The animals were fed freely with standard laboratory chow and were housed in a constant temperature (22 ± 1℃) and humidity (65–70%) environment with a 12-h light/dark cycle. After completing the experiments, animals were anesthetized (1.5–2% isoflurane) and sacrificed by cervical dislocation.

#### AngII-Induced Model

Only male mice were studied because female mice have a low incidence of AngII-induced AAA [11]. Male apoe-/- mice aged 8 weeks were anesthetized with isoflurane, and a mini-osmotic pump (Alzet model 2004) containing AngII (Sigma, USA) was implanted subcutaneously into the mice. AngII was released at a rate of 1000 ng/kg/min for 4 weeks [12]. A vessel diameter exceeding 50% of the normal diameter indicates the successful construction of the AAA mouse model [1] The mice were then randomly assigned to two groups, with the TMAO group receiving drinking water containing 0.12% TMAO for 4 weeks [13] and the control group receiving normal drinking water. Saline-containing mini-osmotic pumps were implanted into an additional 10 apoe-/- mice that were then randomly divided into TMAO and control groups. All mice received high-fat diets (3% cholesterol, 0.2% bile salts, 20% lard, 10% white sugar, and high-protein feed). Four weeks later, the mice were sacrificed, and the abdominal aortic segments were collected for immunohistochemistry, western blots, (elastin van Gieson) EVG, and morphological analysis.

#### CaCl_2_-Induced Model

Male C57BL/6 mice aged 8 weeks were anesthetized as described above, and a midline incision was made along the abdomen to expose the abdominal aorta. Gauze soaked with 0.5 mol/l CaCl_2_ was placed directly on the outer membrane of the lower abdominal aorta for 15 min [14]. The mice were then assigned to two groups after modeling. The TMAO group was continuously fed drinking water containing 0.12% TMAO for 4 weeks, while the control group received the same dose of normal drinking water. Cotton gauze with NaCl (0.9%) was used for the sham operation after exposing the abdominal aorta in another 10 C57BL/6 mice that were then randomly assigned to a TMAO group and a control group. All mice received a normal diet. After 4 weeks, the mice were sacrificed, and the abdominal aortic segments of the mice were collected for related analyses.

### Histological Analysis

After 4 weeks of modeling, the mice were sacrificed. The blood was washed out by perfusion of the left ventricle with normal saline. The vessels from the aortic arch to the iliac artery segment were dissected and fixed in 4% formalin or frozen at − 80 °C. The formalin-fixed abdominal aortic segments were gradually dehydrated and embedded in paraffin, and 5-µm sections were prepared for hematoxylin and eosin (H&E) and elastin van Gieson (EVG) staining. Micrographs were analyzed by two blinded investigators using Image-Pro Plus software.

### Immunohistochemistry Staining

Paraffin-embedded tissues were used to analyze the expression of the matrix metalloproteinases (MMP) MMP2 and MMP9 and p16 and p21. The levels of p16, p21, MMP2, and MMP9 in the media were analyzed by determining the optical density of stained areas of the aortic wall. The primary antibodies used in the experiments were MMP2 (Abcam, ab97779), MMP9 (Abcam, ab38898), p16 (Abcam, ab51243), and p21 (Abcam, ab109520). ImageJ software was used to analyze the images by two blinded investigators.

### Western Blotting

The Western blotting procedure was as previously described ^15^. The primary antibodies were MMP2 (Abcam, ab97779), MMP9 (Abcam, ab38898), p16 (Abcam, ab51243), and p21 (Abcam, ab109520). Proteins were quantified by ImageJ software.

### SA-β-Gal Activity Assay

Cells were grown to 70 to 80% confluence in 6-well plates, after which the medium was removed and the cells were washed once in PBS and fixed in 1 ml β-gal staining fixative at room temperature for 15 min. Cells were then washed (3 × 3 min washes in PBS) before the addition of 1 ml of the β-gal stain working solution (10 µl solution A, 10 µl solution B, 930 µl solution C, and 50 µl X-Gal solution) to each well and incubated overnight at 37 °C. Cells were examined under light microscopy with green-stained cells identified as SA-β-gal-positive.

### Elastic Fiber Staining

Paraffin sections were stained with a commercial kit (Solarbio, G1593) according to the manufacturer’s instructions to observe the degradation of the elastic fibers. A standard score was used for the semiquantification of elastin degradation: score 1, no degradation; score 2, mild degradation; score 3, severe degradation; score 4, aortic rupture 16] ^16^.

### Immunofluorescent Staining for ROS Detection

The frozen sections were restored to room temperature, the excess liquid was removed, and the objective tissue was marked with a liquid blocker pen. The sections were then incubated with spontaneous fluorescence quenching reagent for 5 min and washed under running water for 10 min. ROS staining solution was added to the marked area and incubated at 37 °C for 30 min in the dark. After washing (3 × PBS), the nuclei were counterstained with DAPI for 10 min at room temperature in the dark. After washing (3 × PBS), sections were mounted using an anti-fade mounting medium. The slides were then examined under fluorescence microscopy, and images were recorded.

### Cell Culture

Mouse aortic smooth muscle cells (SMCs) were cultured in Dulbecco’s Modified Eagle Medium with 10% fetal bovine serum, streptomycin (100 U/ml), and penicillin (100 U/ml), purchased from Beinart Biotech. The cells were grown for 3–6 generations and reached 70 ~ 80% confluency for the experiment. Four SMC groups were established: (1) control group (only SMC medium); (2) TMAO group (SMC medium with 1 µM TMAO for 24 h); (3) AngII group (SMC medium with 1 μmol/l AngII for 24 h); and (4) AngII + TMAO group (SMC medium with 1 μmol/l AngII plus 1 µM TMAO for 24 h). The AngII + TMAO group was also treated with different concentrations of TMAO (100 nM, 1 µM, and 5 µM) for further analysis. The TMAO was purchased from Sigma-Aldrich (317,594; St Louis, MO, USA).

### Serum TMAO Detection

The serum TMAO level was determined by enzyme-linked immunosorbent assay (ELISA) using a TMAO kit (GEN-VIEW, Hong Kong, China) [17].

### Aneurysm Measurement

All mice were sacrificed after 4 weeks, and the maximum diameters of the abdominal aortae were measured as previously described [18].

### Statistical Analysis

For all statistical tests, a *P* value of < 0.05 was considered statistically significant, and all tests were two-tailed. GraphPad Prism (version 8.02) was used for all analyses. Graphs depict mean ± SD. One-way ANOVA and Student’s *t* test were used for comparisons between multiple groups and two groups, respectively. The two-way ANOVA test was used for two treatments. The D’Agostino-Pearson omnibus normality test or Shapiro–Wilk normality test was used to determine whether the data satisfied the condition of normal distribution. *P* < 0.05 was considered significant (*P < 0.05, **P < 0.01, ***P < 0.001, ****P < 0.0001, ns = not significant).

## Results

### TMAO Enhances AngII-Induced AAA Development in Mice

**Fig. 1:**
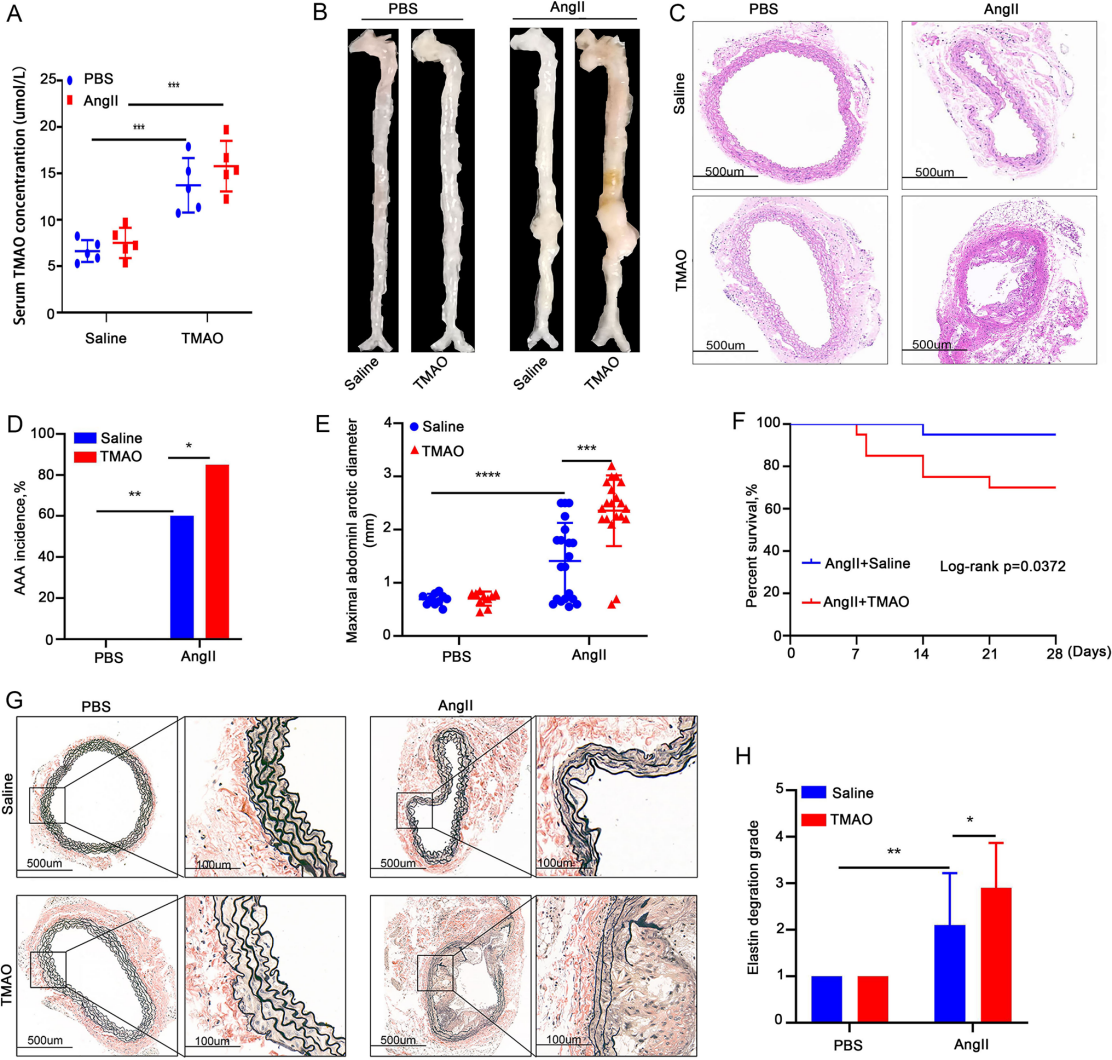
TMAO increases AAA development in AngII-induced mouse models. **A** The serum TMAO level in TMAO-treated mice and in saline-treated mice. **B** Abdominal aorta after saline and TMAO treatment. **C** H&E micrographs from each group (scale bars = 500 µm). **D**–**E** AAA incidence (D) and analysis of maximal abdominal aortic diameters in each group (E). **F** The survival curve of mice after saline and TMAO treatment. **G** EVG staining in the suprarenal aorta after AngII or PBS treatment (scale bars, 500 µm, and 100 µm). **H** Analysis of elastin degradation scores from each group (*n* = 10 per PBS group and *n* = 20 per AngII group)

The serum TMAO level was significantly higher in TMAO-treated mice than in saline-treated mice, which indicated that the use of TMAO supplementation in the drinking water was successful (Fig. 1A). No AAA formation was observed in the PBS group after saline or TMAO treatment (Fig. 1B, C). The HE and EVG staining results showed both normal morphology and normal numbers of elastin layers in the abdominal aorta of saline-treated mice (Fig. 1C, G). Mice that received AngII infusion had an AAA incidence of 85% (17/20) in the TMAO group, which was significantly higher than the 60% (12/20) in the saline group. Meanwhile, 30% (6/20) of mice in the TMAO group died of AAA rupture, while the mortality rate in the saline group was only 15% (3/20) (Fig. 1D, F). The presence of abdominal aortic rupture was defined by the observation of a blood clot in the retroperitoneal cavity [11]. Additionally, compared with the saline group, the mice in the TMAO-treated group had significantly larger AAA diameters and higher vascular elastin fiber degradation scores (Fig. 1E, F, G, H). These results indicated that TMAO promotes the formation of AngII-induced AAA.

### TMAO Augments AngII-Induced AAA Pathology in Mice

**Fig. 2:**
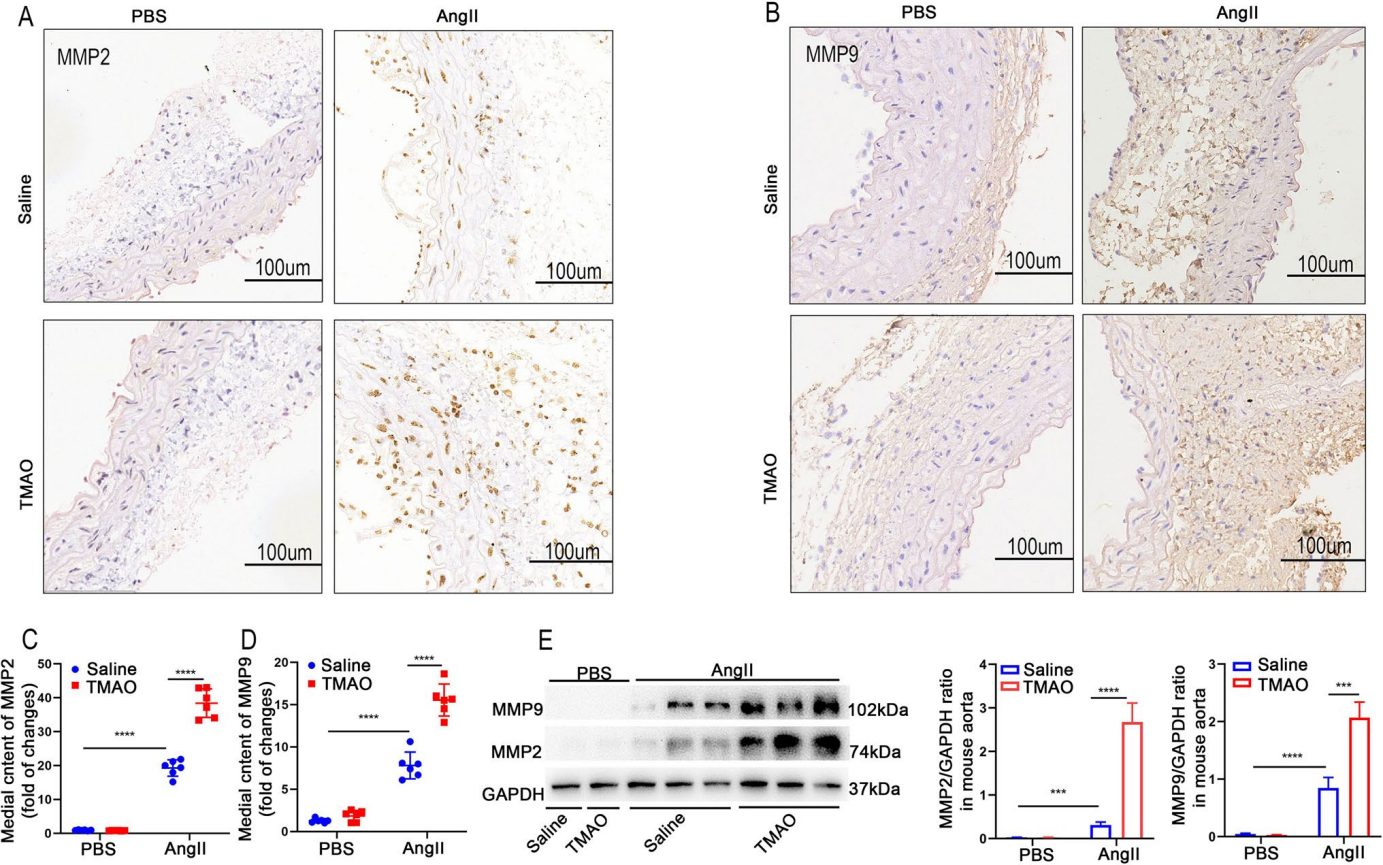
TMAO exacerbates AngII-induced pathological changes associated with AAA in mice. Mice were infused with AngII or PBS for 28 days. **A** and **B** MMP2 and MMP9 immunostaining in the abdominal aorta after AngII or PBS treatment. Bar = 100 μm. **C** and **D** Densitometry of MMP2 (C) and MMP9 (D) content in PBS- and AngII-treated mice (*n* = 6). **E** Western blotting and analysis of MMP9 and MMP2 in the abdominal aorta (*n* = 3)

MMPs play crucial roles in AAA formation [19]. Therefore, the study investigated MMP2 and MMP9 expression in the abdominal aortae of each group of mice by western blot and immunohistochemical staining. MMP2 and MMP9 expression in the abdominal aortic segments of mice in the AngII-treated group was significantly greater than that in the PBS group, in which TMAO further increased the expression of MMP2 (Fig. 2A, C, E) and MMP9 (Fig. 2B, D, E) in AngII-treated mice. In contrast, no increase in MMP2 and MMP9 was observed in the PBS group after TMAO treatment (Fig. 2A–E). These results indicated that TMAO aggravated AngII-induced MMP2 and MMP9 expression in the abdominal aorta, exacerbating the formation of AAA.

### TMAO Promotes CaCl_2_-Induced AAA Development in Mice

**Fig. 3:**
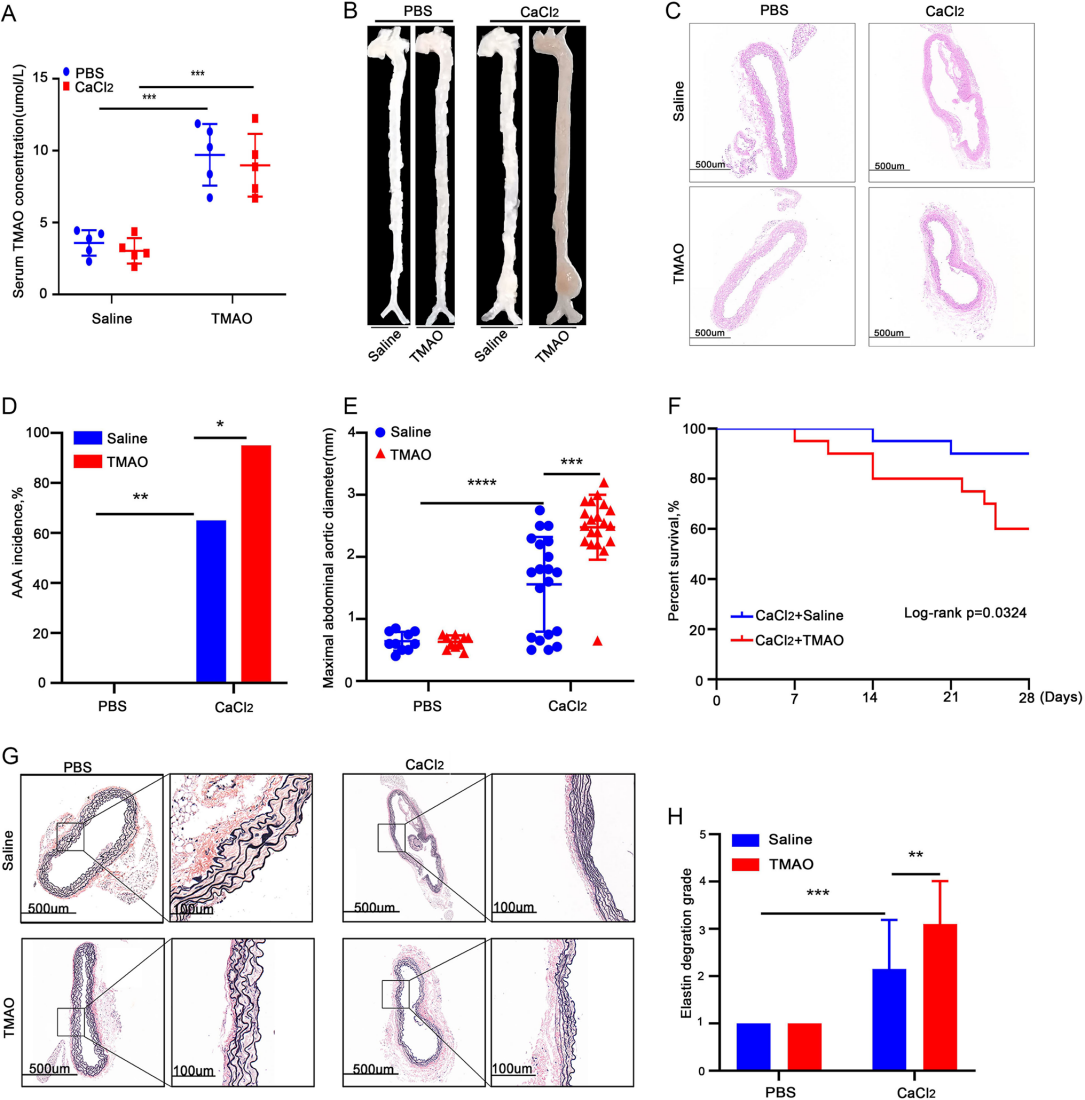
TMAO promotes CaCl_2_-induced AAA development in mice. **A** The serum TMAO level in TMAO-treated mice and in saline-treated mice. **B** Morphological images of each group of mice; **C** H&E-stained sections from each group (scale bars = 500 µm). **D**–**E** AAA incidence (D) and maximal abdominal aortic diameters of each group (E). **F** Survival curves of mice after saline and TMAO treatment in the CaCl_2_-induced AAA and control groups. **G** EVG-stained suprarenal aortae from PBS- and CaCl_2_-induced mice (scale bars, 500 µm, and 100 µm). **H** Analysis of elastin degradation scores of each group (*n* = 10 per PBS group and *n* = 20 per CaCl_2_ group)

To further investigate the role of TMAO in AAA formation, the study used the calcium chloride–induced AAA model for experiments. The study first measured the levels of TMAO in the serum of both the saline- and TMAO-treated groups of mice and confirmed the success of the TMAO administration (Fig. 3A). The results showed no AAA formation in the PBS group (Fig. 3B). HE and EVG staining showed no significant changes in the maximum diameter of the abdominal aorta and vascular elastic fibers in TMAO-treated versus saline-treated mice (Fig. 3C, G). In the CaCl_2_ group, TMAO treatment significantly increased the maximum diameter of AAA (Fig. 3E.) Meanwhile, the incidence of AAA (19/20) and the mortality rate of mice in the TMAO group (8/20) were significantly higher than those in the PBS group (13/20 and 2/20, respectively) (Fig. 3D, F). In addition, the degradation score of vascular wall elastin fibers in the TMAO group was higher than that of the saline group after CaCl_2_ stimulation (Fig. 3G, H), indicating that the degradation of vascular wall elastin fibers in the TMAO group was more severe. These results showed that TMAO promotes CaCl_2_-induced AAA development in mice.

### TMAO Promotes the Expression of MMP in Mice with CaCl_2_-Induced AAA

**Fig. 4:**
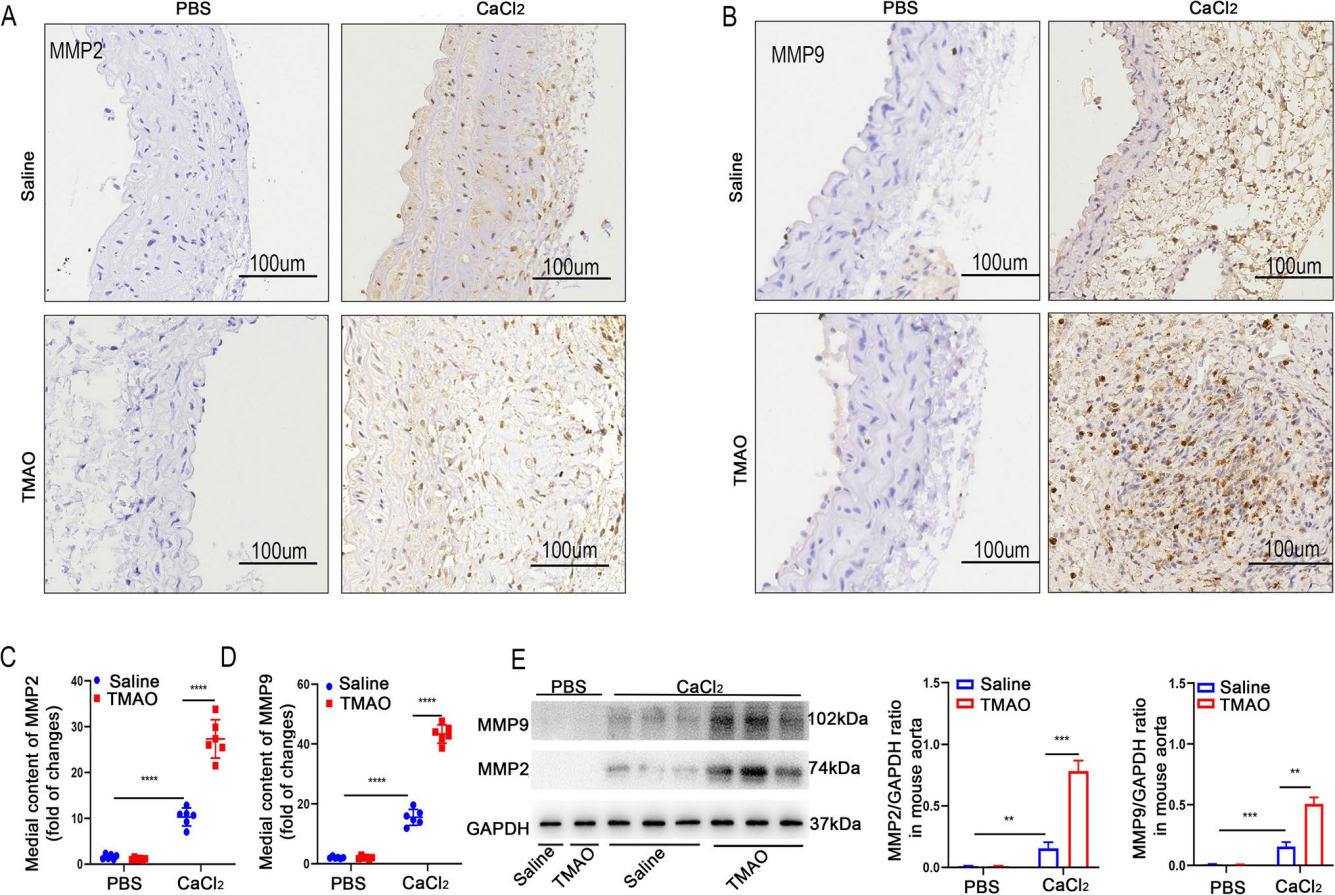
TMAO promotes AAA-related pathology in CaCl_2_-induced mouse models. **A** and **B** MMP2 and MMP9 immunostaining in abdominal aortae from mice after CaCl_2_ and PBS treatment. Bar = 100 μm. **C** and **D** Densitometry of MMP2 (C) and MMP9 (D) contents in mice after CaCl_2_ or PBS treatment (*n* = 6). **E** Western blotting and analysis of MMP9 and MMP2 in mice after CaCl_2_ or PBS treatment

To verify whether TMAO could promote the expression of MMP in AAA, the expression of MMP2 and MMP9 in the abdominal aortae of CaCl_2_-induced AAA model mice was measured. The immunohistochemical results showed that CaCl_2_ treatment significantly increased the expression of both MMPs compared to the PBS group, where TMAO further promoted CaCl_2_-induced MMP2 and MMP9 expression (Fig. 4A–D). Next, the MMP2 and MMP9 concentrations in the abdominal aortae were determined by western blot (Fig. 4E), and the findings were consistent with the immunohistochemistry results. These results suggested that TMAO promotes the expression of MMP in AAA, which leads to the development of AAA.

### TMAO Increases Cellular Senescence and ROS Accumulation in Mice

**Fig. 5:**
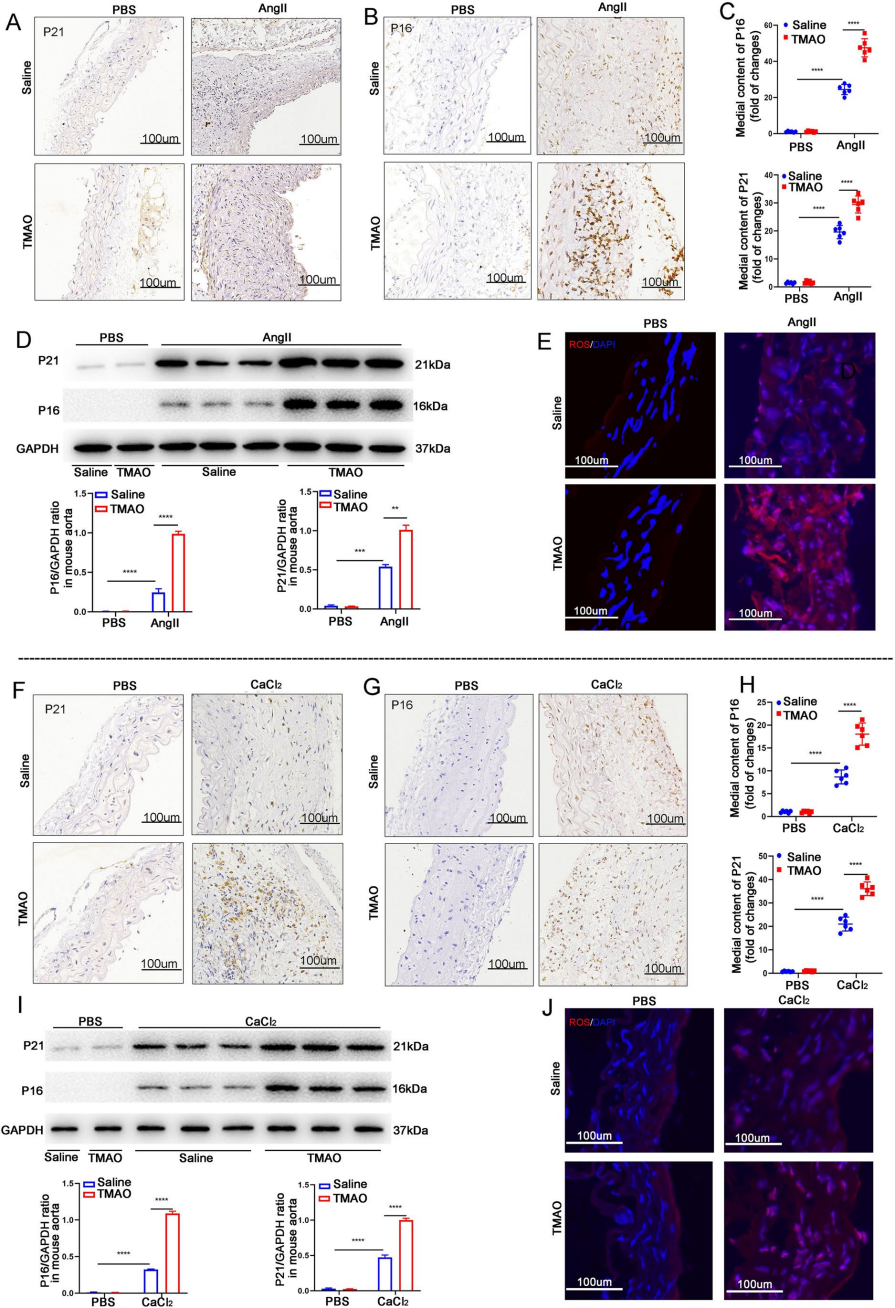
TMAO increases the levels of senescence-related markers and ROS accumulation in mice. **A** and **B** p16 and p21 immunostaining in abdominal aortae from mice after AngII or PBS treatment. Bar = 100 μm. **C** Densitometry of p16 and p21 contents (*n* = 6). **D** Western blotting and analysis of p16 and p21 in mice after AngII or PBS treatment (*n* = 3). **E** The level of ROS in the abdominal aortas of AngII- and PBS-infused mice was evaluated by dihydroethidium (DHE) staining (scale bars, 100 µm). **F** and **G** p16 and p21 immunostaining in abdominal aortae from mice after CaCl_2_ or PBS treatment. Bar = 100 μm. **H** Densitometry of p16 and p21 contents after CaCl_2_ or PBS treatment (*n* = 6). **I** Western blotting and analysis of p16 and p21 in mice after CaCl_2_ or PBS treatment (*n* = 3). **J** The level of ROS in the abdominal aortas of CaCl_2_- and PBS-infused mice was evaluated by dihydroethidium (DHE) staining (scale bars, 100 µm)

It has been reported that senescence is closely associated with AAA formation, characterized by the upregulation of p16 and p21 protein expression [1, 20] . Therefore, this study investigated p16 and p21 expression in the abdominal aortae of AAA mouse models. In the AngII-induced models, immunohistochemistry showed that AngII increased both p16 and p21 expression, while TMAO promoted further AngII-induced expression of the proteins (Fig. 5A–C). Next, the levels of p16 and p21 in the abdominal aortic segments of the mice were determined by western blot, and the findings were consistent with the immunohistochemistry results (Fig. 5D). As reactive oxygen species (ROS) play a crucial role in AAA formation [21], ROS levels in the vascular wall with or without TMAO in the AngII-induced mice were examined. As shown in Fig. 5E, TMAO significantly promoted AngII-induced ROS accumulation in mice. Meanwhile, both the immunohistochemistry and WB results in the CaCl_2_-induced AAA model showed that TMAO also promoted CaCl_2_-induced expression of p16 and p21 (Fig. 5F–I). TMAO also promoted ROS accumulation in CaCl_2_-induced AAA mice (Fig. 5J). These results indicate that TMAO promotes vascular senescence and ROS accumulation in the mouse AAA model, which is likely to be the mechanism by which TMAO promotes AAA development.

### TMAO Increases AngII-Induced Senescence and ROS Accumulation *In Vitro*

**Fig. 6:**
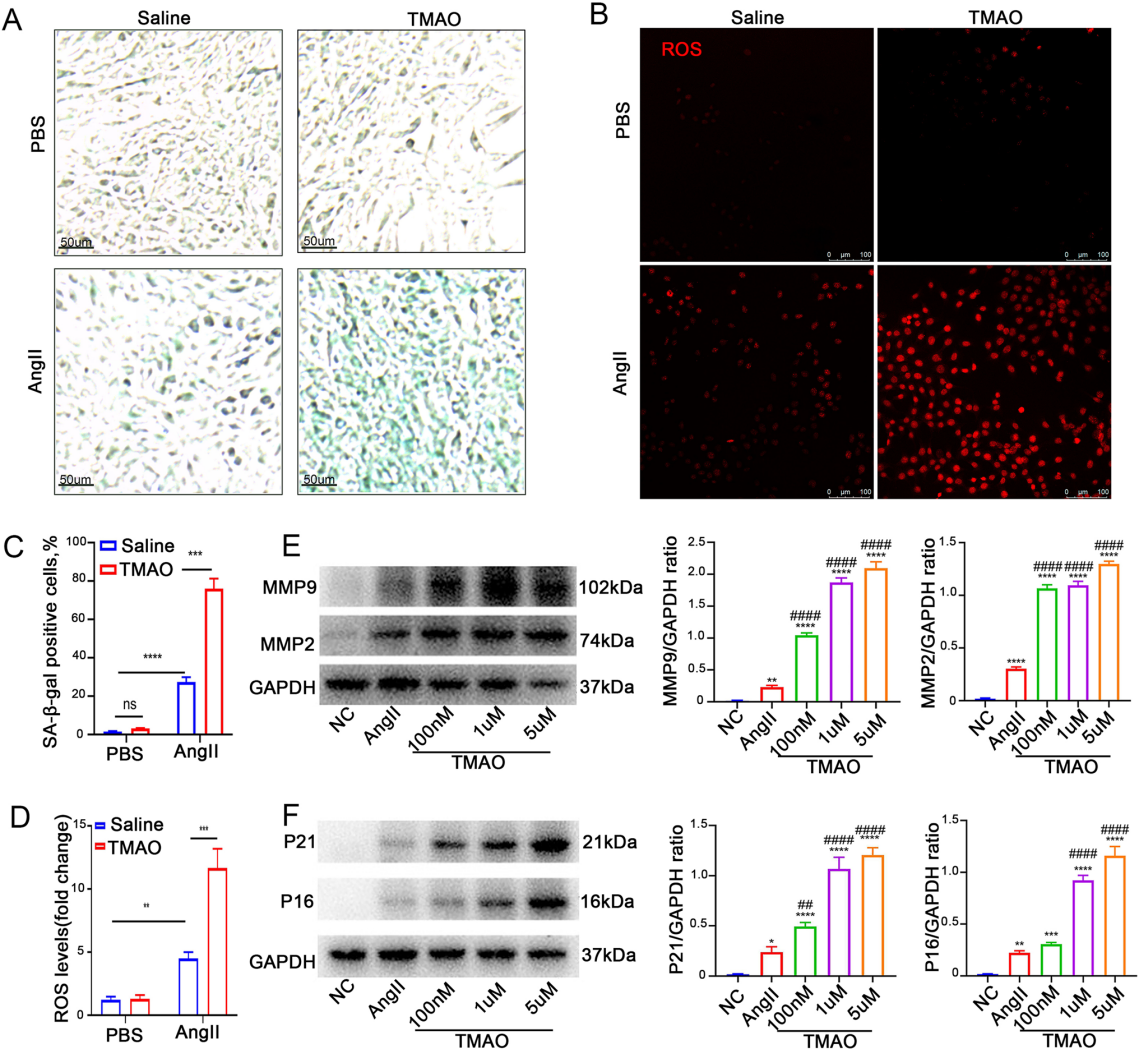
TMAO increases AngII-induced cellular senescence and ROS accumulation in vitro. **A**, **C** SA-β-gal staining was used to assess SMC senescence; the green-stained cells were senescent cells (*n* = 3). The positive and negative controls were treated with AngII and PBS, respectively. TMAO groups were treated with AngII (1 μmol/l) and TMAO (100 nM, 1 µM, and 5 µM). **B**, **D** The ROS level was detected by DHE staining and quantified by determining the ratio of DHE-positive cells (*n* = 3). **E** Western blotting and analysis of MMP2 and MMP9. **F** Western blotting and analysis of p21 and p16 (*n* = 3 per group)

To analyze the effects of TMAO on SMCs, SMCs were incubated with AngII and/or TMAO before measuring SA-β activity. SA-β staining showed that AngII significantly increased SA-β activity, and TMAO treatment further promoted this effect (Fig. 6A, C). Interestingly, as Fig. 6B shows, TMAO further exacerbated AngII-induced ROS accumulation in SMCs. Meanwhile, TMAO treatment alone did not appear to substantially affect SMCs (Fig. 6B, D). The expression of AAA-related MMP and senescence-related markers after TMAO treatment was also examined. TMAO promoted the AngII-induced expression of MMP2, MMP9, p16, and p21 in a concentration-dependent manner (Fig. 6E–F). These results suggested that TMAO exacerbates AngII-induced cellular senescence and ROS accumulation, which might be a possible mechanism by which TMAO promotes AAA formation.

## Discussion

This study first found that TMAO promotes AAA development in mice. In addition, TMAO increased AngII-induced smooth muscle cell senescence and ROS accumulation, suggesting that this is involved in the development of AAA. The development of AAA is not a static event but a dynamic and complex pathophysiological progression from minimal dilation to a clinically relevant aneurysm [22]. It has been widely reported that cellular senescence is associated with SMC damage in AAA [23]. In epidemiological studies, age is an independent risk factor for AAA [24]. In addition, cell senescence is aggravated by both extended physiological life and pathological stimuli, including exposure to toxic and proinflammatory factors. Senescent SMCs have a reduced proliferative capacity and are more prone to apoptosis and the development of a proinflammatory phenotype [25]. These phenotypes could explain the reduction and impairment of SMCs during the development of AAA. Additionally, evidence shows that senescent SMCs can be found in AAA patients and AAA mouse models [26].

TMAO is a metabolite produced by gut microbes that has a close association with cardiovascular disease [10]. In recent studies, it has been reported that TMAO is involved in the regulation of cellular senescence and vascular inflammation [27]. Yilang Ke et al. found that elevated circulating TMAO may accelerate endothelial cell senescence and vascular aging by repressing SIRT1 expression, increasing oxidative stress, and activating the p53/p21/Rb pathway [10]. Similarly, Guliang Yang et al. have revealed that TMAO induced vascular inflammation, which was probably associated with the NF-κB/MAPK pathways [28]. These findings all imply that TMAO may contribute to cardiovascular diseases by regulating cellular senescence and vascular inflammation. In the present study, TMAO was observed to promote cell senescence in AAA models of mice and cultured smooth muscle cells, suggesting that TMAO is a risk factor in AAA patients. TMAO is linked with various cardiovascular diseases and may even be the cause of these diseases.

The present study demonstrated for the first time, using both in vivo and in vitro experiments, that TMAO can contribute to AAA development by promoting SMC senescence and ROS accumulation. Smoking, age, family history, male sex, lipid levels, and hypertension have been considered risk factors for AAA [29]. The relationship between gut microbiota metabolites and AAA is a crucial question that needs to be answered. TMAO levels can be measured in the blood of AAA patients on a large scale to validate our observations. In addition, whether TMAO affects the function of other cells during AAA development and its mechanisms at the molecular level needs to be further investigated. Furthermore, this study only focused on the relationship between SMC senescence, ROS accumulation, and AAA formation, and the causal relationship between them needs to be further investigated.

In summary, this study demonstrated a link between TMAO and AAA formation, which may be related to the promotion of SMC senescence and ROS accumulation by TMAO. This provides new insights into the prevention of AAA formation.

## Abbreviations

AAA: Abdominal aortic aneurysm
TMAO: Trimethylamine N-oxide
SMCs: Smooth muscle cells
AngII: Angiotensin II
CaCl_2_: Calcium chloride
CVD: Cardiovascular disease
MMP: Matrix metalloproteinases
ROS: Reactive oxygen species
